# The gametocytocidal efficacy of primaquine in malaria asymptomatic carriers treated with dihydroartemisinin-piperaquine in The Gambia (PRINOGAM): study protocol for a randomised controlled trial

**DOI:** 10.1186/s13063-015-0597-1

**Published:** 2015-03-01

**Authors:** Joseph Okebe, Teun Bousema, Muna Affara, GianLuca DiTanna, Alice C Eziefula, Musa Jawara, Davis Nwakanma, Alfred Amambua-Ngwa, Jean-Pierre Van geertruyden, Chris Drakeley, Umberto D’Alessandro

**Affiliations:** Disease Control & Elimination Theme, Medical Research Council Unit, Fajara, The Gambia; Immunology and Infection Department, London School of Hygiene and Tropical Medicine, London, UK; Department of Infectious Disease Epidemiology, Faculty of Epidemiology and Population health, London School of Hygiene and Tropical Medicine, London, UK; International Health Unit, Faculty of Medicine & Health Sciences, University of Antwerp, Antwerp, Belgium; Department of Disease Control, Faculty of Infectious and Tropical Diseases, London School of Hygiene and Tropical Medicine, London, UK; Department of Public health, Institute of Tropical Medicine, Antwerp, Belgium

**Keywords:** Malaria, Plasmodium falciparum, Asymptomatic infection, Gametocyte, Primaquine, Safety, Efficacy, Elimination

## Abstract

**Background:**

Finding efficacious tools to decrease and interrupt malaria transmission is essential to sustain the gains in malaria control and contain the emergence of artemisinin resistance. Primaquine is effective against *Plasmodium falciparum* gametocytes and recommended for treatment campaigns in (pre-)elimination settings. Safety concerns preclude its use in endemic African countries with variable proportions of glucose-6-phosphate dehydrogenase (G6PD)-deficient individuals. The efficacy of the current recommended dose needs to be evaluated, particularly in individuals with an asymptomatic malaria infection.

**Methods/design:**

This is a four-arm, open label, randomized controlled trial that aims to determine and compare the effect of three different single doses of primaquine combined with dihydroartemisinin-piperaquine, an artemisinin-based combination therapy, on gametocyte carriage in asymptomatic, malaria infected, G6PD-normal individuals. Approximately 1,200 participants are enrolled and followed for 42 days, with the primary endpoint being the prevalence of *Plasmodium falciparum* gametocyte carriage at day 7 of follow-up determined by quantitative nucleic acid sequence based amplification assay. Direct membrane feeding experiments to determine infectiousness to mosquitoes are conducted as a biological secondary endpoint.

**Discussion:**

Sub-Saharan Africa, with a relatively high but poorly characterized G6PD prevalence, could potentially benefit from the use of primaquine to further reduce or interrupt malaria transmission. However, G6PD screening may not be feasible given the cost and difficulties in interpreting test results in terms of risk of haemolysis. Because the haemolytic effect of primaquine is dose-dependent, determining the minimal gametocytocidal and transmission-blocking dose of primaquine is extremely important to help address public health concerns over its safety and validate the efficacy of lower than recommended dosages. By including infectiousness to mosquitoes, the trial provides complementary evidence for the potential of the drug to interrupt transmission to mosquitoes.

**Trial registration:**

ClinicalTrials.gov: NCT01838902 (12 April 2013).

## Background

The scale-up of anti-malaria interventions, including artemisinin-based combination therapy (ACT), and the subsequent decline in many malaria indicators has boosted prospects of local or regional malaria elimination [1]. However, it is evident that additional tools, including drugs effective against sexual (gametocyte) stages and programmes that are targeted at asymptomatic parasite carriage, would be needed if the goal of interrupting *Plasmodium falciparum* malaria transmission were to be achieved.

A number of endemic countries, including some in sub-Saharan Africa, have achieved substantial reductions in their malaria burden [1,2] and are considering the possibility of eliminating malaria within their borders. Artemisinin derivatives are extremely effective against both asexual and early-stage *P. falciparum* gametocytes and less so against mature gametocytes [3,4]. Combining ACTs with primaquine (PQ), an 8-aminoquinoline effective against mature gametocytes, targets all parasite stages and thus can halt the transfer of gametocytes from humans to mosquitoes. In this context, PQ could play an essential role in interrupting transmission but there are concerns, especially over its safety and efficacy, that could preclude its large-scale use in these countries.

The optimal PQ dose used in combination with an ACT against *P. falciparum* gametocytes has been the subject of some discussion. Previously recommended by the World Health Organization as a single 0.75 mg base/kg, it was recently revised to 0.25 mg base/kg as this dose was unlikely to result in serious toxicity, especially in individuals with any glucose-6-phosphate dehydrogenase (G6PD) deficiency variant, while retaining its gametocytocidal effect [5]. Whereas the 0.75 mg base/kg PQ dose had a variable impact on transmission [6,7], the efficacy of the 0.25 mg base/kg has not been evaluated in any recent trial.

A recently concluded dose-ranging trial of PQ plus artemether-lumefantrine in children with uncomplicated malaria showed that a 0.4 mg/kg dose was as efficacious as the 0.75 mg/kg dose in clearing gametocytes [8]. The trial excluded phenotypically G6PD-deficient individuals by fluorescent spot test and observed no clinically relevant haemolysis, although a statistically significant haemolytic effect was observed in phenotypically G6PD-sufficient but genotypically G6PD-deficient individuals receiving 0.75 mg/kg or 0.4 mg/kg PQ [9].

The dynamics of gametocyte carriage may differ considerably between symptomatic and asymptomatic infections and these differences in gametocyte dynamics may explain why previous studies in Africa have reached different conclusions on the added value of PQ in symptomatic and asymptomatic infections [7,10]. Although the acute phase of clinical malaria attacks have been associated with a subsequent wave of gametocytes that may persist longer after treatment [11], asymptomatic individuals typically have lower densities of both asexual and sexual parasites yet potentially have more mature gametocytes as a result of the long duration of asymptomatic parasite carriage [12]. Because asymptomatic infections typically comprise the majority of malaria infections in populations [13], studies in asymptomatic parasite carriers are needed to provide complementary evidence for the efficacy and safety of even lower PQ doses.

This study combines gametocyte carriage measured using molecular detection methods [10,14] with a complementary biological endpoint of gametocyte infectivity in a sub-group of study subjects to assess the transmission blocking effect of PQ [15].

### Trial objectives and endpoints

The trial aims to determine and compare the effect of three different single doses of PQ combined with an ACT, dihydroartemisinin-piperaquine (DHA-PPQ), on gametocyte carriage in asymptomatic, malaria-infected, G6PD-normal individuals. In addition, it will determine and compare the effect of the above combinations on change in haemoglobin concentration during follow-up, infectiousness to mosquitoes, parasite clearance time and cure rate.

The main trial endpoint is the prevalence of *P. falciparum* gametocyte carriage at day 7 of follow-up determined by quantitative nucleic acid sequence based amplification assay (QT-NASBA).

Other endpoints include: 1) the prevalence of *P. falciparum* gametocyte carriage, determined by QT-NASBA, during other follow-up visits; 2) change in haemoglobin values between enrollment (day 0) and each day of follow-up; 3) infectiousness to mosquitoes on day 7 determined by direct membrane feeding assay (DMFA); 4) proportion of participants with recurrent infections (polymerase chain reaction adjusted and unadjusted) after day 7; and 5) the prevalence of adverse events and serious adverse events.

## Methods

### Design

This is a four-arm, open label, randomized controlled trial in which G6PD-normal asymptomatic *Plasmodium falciparum*-infected individuals identified through population screening are randomized to receive either a complete course of DHA-PPQ alone (control) or a course of DHA-PPQ plus one of three different doses of PQ (intervention): 0.75 mg base/kg, 0.4 mg base/kg and 0.2 mg base/kg, given as a single dose.

### Study area and participant selection

**Figure 1:**
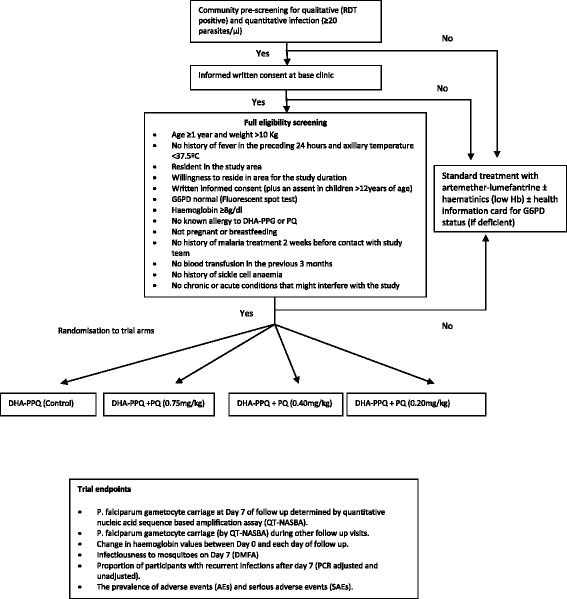
**DHA-PPQ, dihydroartemisinin-piperaquine; DMFA, direct membrane feeding assay; Hb, haemoglobin; G6PD, glucose-6-phosphate dehydrogenase; PCR, polymerase chain reaction; PQ, primaquine; RDT, rapid diagnostic test.**

Participants are recruited from villages around the Medical Research Council’s field stations in the Central and Upper River Regions in The Gambia. Malaria transmission in the country is well described, and characterized by marked seasonality with a peak between September and November [16]. Participants are selected in a two-stage screening process. An initial pre-screening is used to determine infection (rapid antigen test kit) and establish a baseline parasitaemia (microscopy). Individuals aged 1 year and above with parasite density ≥20 parasites/μl are invited to the base clinic the following day where, after a written informed consent, they undergo screening for G6PD status (N Dimopoulos SA, Greece), haemoglobin measurement (Hemocue, Ängelholm, Sweden) and, if eligible, are enrolled (Figure 1). Infected but ineligible persons are treated with a full course of artemether-lumefantrine. In addition, those with haemoglobin counts <8 g/dl receive iron supplements while G6PD-deficient individuals will be counselled on their test results and provided with a notification card indicating their G6PD status which they are advised to present at clinics when seeking medical care.

### Randomisation and blinding

Enrolment into the trial arms follows a randomisation scheme in a 1:1:1:1 ratio using blocks of varying size to ensure a balance in recruitment between the four groups. A randomisation list, generated by the trial statistician using Stata software version 13 (Stata Corp. College Station, TX, USA), is sent to a physician not involved in the trial who puts the codes for the allocation groups into sequentially numbered, opaque envelopes. The envelopes are opened by the clinic team only after the potential participant meets the eligibility criteria.

This is an open-label trial; staff involved in clinical care, including administering the trial drugs, are aware of the assigned groups but those involved in sample processing and data analysis are blinded.

### Treatment and follow-up

All participants in the trial will receive a complete 3-day course of DHA-PPQ based on body weight according to the manufacturer’s instructions. DHA-PPQ is available as both paediatric (dihydroartemisinin 20 mg/piperaquine 160 mg) and adult (dihydroartemisinin 40 mg/piperaquine 320 mg) formulations. Participants in the three intervention arms are randomized to receive a single 0.75 mg/kg, 0.40 mg/kg or 0.20 mg/kg dose of PQ base on day 2 with the third dose of DHA-PPQ. Each tablet of PQ contains 15 mg base and, to ensure the accuracy of doses <15 mg, the tablet will be reconstituted in 15 ml water to achieve a 1 mg/ml concentration and the required dose dispensed using a sterile syringe. A nurse directly observes all treatments and treatment may be repeated once if a participant vomits any of the drugs within 30 minutes of being administered.

Each participant attends eight scheduled follow-up visits on days 3, 7, 10, 14, 21, 28, 35, and 42 for clinical evaluation and blood sampling. Blood samples from a finger prick are collected for gametocyte (QT-NASBA) and asexual parasite clearance (microscopy and polymerase chain reaction) and haemoglobin measurement. Additional tests may be requested during unscheduled visits to the clinic (Table 1). Thick blood films stained with 10% Giemsa for 10 minutes are examined under 1000-fold magnification by trained microscopists. Asexual parasite density is determined by counting the number of asexual parasites per white blood cell (WBC) until 500 WBCs have been counted (assuming a mean WBC count of 8,000/μl) and results presented as parasites/μl.

**Table 1:** **Trial activity on scheduled visit days**

**Day**	**−1**	**0**	**1**	**2**	**3**	**7**	**10**	**14**	**21**	**28**	**35**	**42**
Village screening (RDT+ slide)	X											
Informed consent for the trial		X										
G6PD test	X											
Clinical review (history and examination)		X			X	X	X	X	X	X	X	X
Temperature, pulse		X		X	X	X	X	X	X	X	X	X
Weight		X										
Thick blood film		X	X	X	X	X	X	X	X	X	X	X
Haemoglobin, PCR (filter paper)		X			X	X	X	X	X	X	X	X
Gametocytaemia (QT-NASBA)		X			X	X	X	X		X		X
Adverse events		X	X	X	X	X	X	X	X	X	X	X
Treatment		X	X	X								
Direct membrane feeding assay						X						

G6PD, glucose-6-phosphate dehydrogenase; PCR, polymerase chain reaction; QT-NASBA, quantitative nucleic acid sequence based amplification assay; RDT, rapid diagnostic test.

### Quantitative nucleic acid sequence based amplification assay

The primary endpoint of the trial is the prevalence of gametocytes measured by molecular methods on day 7 of follow-up. The detection and quantification of gametocytes is by real-time QT-NASBA which is based on stage-specific gene expression of Pfs25 mRNA by parasites. QT-NASBA is a high throughput technique suited to detecting mature gametocytes with a sensitivity of 0.01 to 0.1 gametocytes/μl blood [17]. It is based on the activities of three enzymes: Reverse transcriptase, T7 RNA polymerase and RNase H and target-specific forward and reverse primers, which includes a T7 polymerase promoter sequence, for continuous and direct amplification of RNA molecules in a single mixture at an isothermal temperature of 41°C. Real-time gametocyte detection and quantification is by a fluorescent-labelled molecular beacon with a sequence complementary to those of amplified anti-sense RNA molecules.

RNA is extracted manually from 50 μl of blood stored in RNAlater using commercial lysis buffers (Severn Biotech, Kidderminster, Worcestershire, UK) and previously described methods that involve nuclease inactivation and nucleic acid binding [18]. Gametocyte density is calculated in relation to the standard gametocyte stage V dilution series using the time point of amplification at which the fluorescence detecting target amplicons exceed the mean fluorescence of three negative controls + 20 standard deviations [17]. Standard curves for density quantification are generated using trend lines of known quantities of gametocytes from laboratory-cultured NF54 parasite strains.

### Direct membrane feeding assay

To measure biological evidence of gametocyte clearance, a subset of 100 participants from each arm will be randomly selected for direct membrane feeding experiments on day 7 using a published protocol [19]. In summary, 3 ml of blood are collected from the participant’s arm and 500 μl is added to two glass feeders and fed to about 100 locally reared 4- to 5-day-old female *Anopheles gambiae sensu stricto* mosquitoes through an artificial membrane. The setup is maintained at 37°C by a temperature controlled water bath and, after 15 minutes of feeding, fully fed mosquitoes are transferred into a holding cage maintained at 27 to 29°C and sustained on 5% glucose for 7 days while partially fed and unfed ones are destroyed. Mosquitoes that survive to this period are dissected in 0.5% mercurochrome, and their midguts examined for oocyst prevalence and density. The proportion of infected mosquitoes, the number of infected mosquitoes divided by the total number of examined mosquitoes, and the density of oocysts in infected mosquitoes is presented.

### Sample size considerations

The sample size was calculated to detect a difference in gametocyte prevalence on day 7 after treatment, between the control (DHA-PPQ only) and 0.75 mg/kg PQ arm, and between this and lower doses of PQ (0.4 mg/kg and 0.2 mg/kg). Two hypotheses underscore the sample size calculation: 1) treatment with PQ (any dose) and DHA-PPQ results in significantly lower gametocyte carriage compared to treatment with DHA-PPQ alone; and 2) a single dose of 0.4 mg base/kg or 0.2 mg base/kg PQ administered with the last dose of DHA-PPQ has similar gametocytocidal efficacy as a single 0.75 mg base/kg dose.

For the first hypothesis, the underlying assumption is that, although a substantial proportion of asymptomatic carriers also carry gametocytes [20], treatment with an ACT alone would reduce gametocyte prevalence on day 7 to around 18%, and the addition of PQ at 0.75 mg/kg will further reduce the prevalence by 50% (that is, to 9%). A sample size of 300 participants per arm would have 90% power to detect this difference, at the 5% significance level, allowing for 10% of enrolees being non-compliant or lost to follow-up.

In the second hypothesis, assuming a prevalence of 9% in the standard PQ arm, 300 participants would be sufficient, with 90% power, to detect a confidence interval around the prevalence in the lower dose PQ arm below 16% and 80% power to detect that this confidence interval is entirely below 15%.

### Statistical analysis plan

Analysis of efficacy will be on the basis of intention-to-treat. Gametocyte densities will be compared between treatment groups using one-way analysis of variance on log-transformed gametocyte densities; gametocyte carriage during follow-up will be estimated by calculating for each arm the area under the curve (AUC) of both the gametocyte density and gametocyte prevalence against time. The AUC between treatment groups will be compared using non-parametric tests. The Kaplan-Meier estimator will be used to compare gametocyte clearance times between study arms for individuals who are gametocyte positive by QT-NASBA at enrolment.

For the non-inferiority comparison between the PQ arms, the 95% confidence around the gametocyte prevalence in the PQ 0.4/0.2 mg/kg arms at day 7 will be compared to the non-inferiority limit determined by the prevalence in the PQ 0.75 mg/kg arm and delta.

For the DMFA, the proportion of infectious individuals will be compared between arms using chi-squared statistics. The proportion of infected mosquitoes will be compared between groups using mixed models to account for clustering within cages fed on blood from the same individual.

### Data entry and validation

Clinical data will be captured from pre-tested medical records and relevant fields entered onto an electronic case report form base created in Openclinica (OpenClinica LLC, Waltham, Ma, USA). Data will be single entered with source-data verification done for each entry. Laboratory-generated (DMFA and QT-NASBA) data will be double entered onto a Microsoft Access database (Microsoft Corp., Richmond, WA, USA) and linked to the clinical data after validation.

### Ethical approval

The trial was approved by The Gambia Government/MRC Joint Ethics Committee (SCC 1321) on 13 March 2013.

## Discussion

Local and regional malaria elimination is gaining acceptance as a means to sustain the gains made in reducing the malaria burden and limiting/preventing the spread of artemisinin resistance. However, achieving these objectives would require a fresh perspective on the necessary tools and applications.

Although individuals with clinical disease due to asexual *P. falciparum* infection also carry gametocytes in variable amounts [21], asymptomatic infections are believed to play an important role across all levels of malaria transmission and therefore targeting only clinical cases would miss the large human reservoir of infection [12]. This concept is supported by evidence from empirical studies and mathematical models [22,23], and the combination of an ACT with PQ is recommended in places where (pre-)elimination is being considered [24].

In asymptomatic infections, parasite density (both asexual forms and gametocytes) are much lower than in clinical cases. However, one model estimate predicts that these low-density infections, even when undetectable by microscopy, may still be infectious to mosquitoes [25]. Parasite densities are estimated by counting parasites against a given number of WBCs, usually 200 WBCs, and then assuming 8,000 WBCs per μl [26]. Nevertheless, when parasite densities are <100/μl, a higher sensitivity may be needed [26] and this can be done by increasing the number of WBCs against which the number of parasites are counted, usually 500. Given the short time available to recruit participants into the trial, counting parasites against 500 WBCs is extremely challenging.

As the trial aims to determine the lowest gametocytocidal PQ dose, it was necessary to include individuals with microscopically detectable infections. The threshold of >20 parasites/μl as an inclusion criterion was chosen as a proxy to increase the likelihood for gametocytaemia [11]. Including carriers of submicroscopic infection would not have been feasible given the need to determine their eligibility in a relatively short time. Blood slides can be re-read later and the number of WBCs per slide increased for higher sensitivity [27].

The potential for inducing haemolysis is probably the main reservation for the widespread use of PQ. In sub-Saharan Africa, the overall prevalence of G6PD deficiency is considered to be high [28]. A recent survey in The Gambia showed that the prevalence of the 202A- mutation, reportedly the most common in sub-Saharan Africa, was 1.8% while the phenotype prevalence was 6.4% overall; 7.8% in males and 4.9% in females [29]. Such low numbers may suggest a low risk but does not eliminate it entirely as individuals with “normal” G6PD status have developed haemolysis [30]. A trial to evaluate the tolerability and safety of increasing doses of PQ in combination with an ACT in G6PD-deficient males with an asymptomatic *P. falciparum* malaria infection has recently been initiated (NCT02174900) and the results would complement those of this trial.

As the haemolytic effect of PQ is dose-dependent, determining the minimal gametocytocidal dose is considered an extremely important first step in addressing public health concerns related to the safety and efficacy of PQ as summarized in a recent systematic review [31]. Since the initial trial protocol was developed, a single 0.25 mg base/kg dose of PQ combined with an ACT is now recommended as sufficiently safe and gametocytocidal to be used without screening for G6PD deficiency [5]. However, the evidence for this dose may be considered inadequate by contemporary standards.

The optimization of the PQ dosing regimen therefore represents an important knowledge gap and research priority [32] as it provides a “new/old” tool for malaria (pre-)elimination. The recently concluded dose-finding trial in children aged 1 to 10 years with uncomplicated falciparum malaria and normal G6PD enzyme function provides encouraging complementary information to support research in G6PD-deficient individuals [8]. Also, the haematological consequences may be less manifest in asymptomatic carriers as the malaria-related haemolysis would be less pronounced.

By evaluating infectiousness to mosquitoes, the trial contributes a biological endpoint - the evidence for transmission reduction or complete transmission blockade to mosquitoes that is ultimately relevant for public health policy making.

## Trial status

Recruitment started in August 2013 with trial results expected in December 2015.

## Abbreviations

ACT: artemisinin-based combination therapy
AUC: area under the curve
DHA-PPQ: dihydroartemisinin-piperaquine
DMFA: direct membrane feeding assay
G6PD: glucose-6-phosphate dehydrogenase
PQ: primaquine
QT-NASBA: quantitative nucleic acid sequence based amplification assay
WBC: white blood cell
